# Fabrication of nanostructured lipid carriers ocugel for enhancing Loratadine used in treatment of COVID-19 related symptoms: statistical optimization, *in-vitro*, *ex-vivo*, and *in-vivo* studies evaluation

**DOI:** 10.1080/10717544.2022.2115164

**Published:** 2022-09-05

**Authors:** Rehab Abdelmonem, Inas Essam Ibrahim Al-Samadi, Rasha M. El Nashar, Bhaskara R. Jasti, Mohamed A. El-Nabarawi

**Affiliations:** a Department of Industrial Pharmacy, College of Pharmaceutical Sciences and Drug Manufacturing, Misr University for Science and Technology, Giza, Egypt; b Department of Chemistry, Faculty of Science, Cairo University, Giza, Egypt; c Department of Pharmaceutics and Medicinal Chemistry, Thomas J. Long School of Pharmacy and Healthy Science-Pacific University, Stockton, CA, USA; d Department of Pharmaceutics and Industrial Pharmacy, Faculty of Pharmacy, Cairo University Giza, Giza, Egypt

**Keywords:** Loratadine (LORA), reducing of COVID-19 ocular symptoms, nanostructured lipid carriers, confocal laser scanning microscopy (CLSM), Draize test, pharmacokinetic studies

## Abstract

Loratadine (LORA), is a topical antihistamine utilized in the treatment of ocular symptoms of COVID-19. The study aimed to develop a Loratadine Nanostructured Lipid Carriers Ocugel (LORA-NLCs Ocugel), enhance its solubility, trans-corneal penetrability, and bioavailability. full-factorial design was established with 2^4^ trials to investigate the impact of several variables upon NLCs properties. LORA-NLCs were fabricated by using hot melt emulsification combined with high-speed stirring and ultrasonication methods. All obtained formulae were assessed in terms of percent of entrapment efficiency (EE%), size of the particle (PS), zeta potential (ZP), as well as *in-vitro* release. Via using Design Expert® software the optimum formula was selected, characterized using FTIR, Raman spectroscopy, and stability studies. Gel-based of optimized LORA-NLCs was prepared using 4% HPMC k100m which was further evaluated in terms of physicochemical properties, Ex-vivo, and In-vivo studies. The optimized LORA-NLCs, comprising Compritol 888 ATO^®^, Labrasol^®^, and Span^®^ 60 showed EE% of 95.78 ± 0.67%, PS of 156.11 ± 0.54 nm, ZP of −40.10 ± 0.55 Mv, and Qh6% of 99.67 ± 1.09%, respectively. Additionally, it illustrated a spherical morphology and compatibility of LORA with other excipients. Consequently, gel-based on optimized LORA-NLCs showed pH (7.11 ± 0.52), drug content (98.62%± 1.31%), viscosity 2736 cp, and Q12% (90.49 ± 1.32%). LORA-NLCs and LORA-NLCs Ocugel exhibited higher ex-vivo trans-corneal penetrability compared with the aqueous drug dispersion. Confocal laser scanning showed valuable penetration of fluoro-labeled optimized formula and LORA-NLCs Ocugel through corneal. The optimized formula was subjected to an ocular irritation test (Draize Test) that showed the absence of any signs of inflammation in rabbits, and histological analysis showed no effect or damage to rabbit eyeballs. C_max_ and the AUC_0–24_ were higher in LORA-NLCs Ocugel compared with pure Lora dispersion-loaded gel The research findings confirmed that NLCs could enhance solubility, trans-corneal penetrability, and the bioavailability of LORA.

## Introduction

Since the emergence of the coronavirus in Wuhan China in 2019 many ocular manifestations of (COVID-19) have prompted investigations into its consequence and impact on humans (Soltani et al., 2022). A meta-analysis and systematic review involving 38 studies with an entire 8,219 COVID-19 patients were established based on the current evidence, the studies determined the prevalence of ocular symptoms have more exaggerated in patients with severe COVID-19 systemic symptoms with acute symptoms of conjunctivitis, ocular irritation, redness, foreign body sensation, soreness, eyelid swelling, tearing, mucoid discharge, congestion and chemosis (88.8%) among COVID-19 patients (Hoogewoud et al., 2021; Nasiri et al., 2021).

Topical anti-histamines represent one of the treatments of ocular findings of COVID-19 reported in the adult population (Danthuluri & Grant, 2020). LORA is a selective inverse agonist of peripheral histamine H1-receptors is used to relieve the symptoms associated with conjunctivitis (Tang et al., 2022a). Moreover, LORA also plays a crucial role as an antimicrobial agent (Zheng et al., 2022). Despite the high activity of LORA, its poor solubility frequently leads to poor absorption and consequently to low concentrations of the drug in the target tissues, thus representing a significant obstacle for the pharmaceutical industry (McGuckin et al., 2022).

Although the oral route of administration is preferred for many drugs, it can be problematic for the drugs belonging to Biopharmaceutical Classification System II basic (BCS IIb) such as LORA where the drug exhibits poor solubility in aqueous solutions, though high permeability and dissolution is a speed-limiting step of absorption (Zhang et al., 2021).

Nanostructured lipid carriers (NLCs) are considered the modified generation of solid lipid nanoparticles, incorporating both liquid and solid lipid as an oil matrix (Hosny et al., 2022). NLCs are better than other conventional nanocarriers in improving the solubility, drug loading of lipophilic drugs, and trans-corneal penetrability, which provides a property of controlled and prolonged release of drug (Mishra et al., 2016). It is fabricated with biodegradable lipids and offers greater solubility and bioavailability (Gilani et al., 2021b). Most topical treatments available on the market are aqueous emulsions or solutions of the drug, however, such topical treatments have some drawbacks, as the rapid mixing of eye drops with the lacrimal fluid reducing drug’s half-life on the ocular surface (estimated to be ∼4 min) before being removed from the eye through nasolacrimal drainage and the conjunctival vasculature. As such, there is significant interest in identifying methods to improve the residence time of the drug on the ocular surface and thus increase its bioavailability (Dave et al., 2021). Mucoadhesive ocugel drug delivery systems obtained their mucoadhesive properties from HPMC K100 polymer which has a relatively high viscosity, thus, extending retention time in the cornea and conjunctival sac, and sustaining drug release despite some physiological responses for example, the nasolacrimal draining reflex and ocular blinking (Mamatha et al., 2022).

This study aimed to formulate and evaluate of LORA-NLCs and evaluate their ability to enhance solubility and trans-corneal penetrability of LORA, additionally study the ocugel impact on enhancing ocular residence time and bioavailability of LORA.

## Material and methods

### Materials

Loratadine^®^ (LORA) was a gift from SEDICO Pharmaceutical Co., Egypt. Compritol 888 ATO^®^ (glyceryl behenate), Labrafil^®^ 2125 (linoleoyl polyoxyl-6 glycerides), and Labrasol^®^ (caprylocaproyl macrogol-8 glycerides) were purchased from Gattefosse (St-Priest, France). Sorbitan monostearate (Span^®^60) was purchased from Oxford Laboratories Pvt. Ltd. Pharmaceutical Company, Mumbai, India. Brij^®^35 (Polyoxyethylene lauryl ether) was supplied by Sigma-Aldrich (Spain) and Aladdin Chemistry Co., Ltd. (Shanghai, China), Hydroxypropyl methylcellulose HPMC k100 was purchased from Lobachemi, Pvt Ltd. Mumbai, India. All utilized analytical grade solvents, chemicals, and reagents were obtained from authorized sources.

### Methods

#### Preparation of Loratadine loaded nanostructured lipid carriers (LORA-NLCs)

LORA-NLCs were prepared by using hot melt emulsification combined with high-speed stirring and ultrasonication methods (Czajkowska-Kośnik et al., 2021), where LORA (10 mg) was combined with solid lipid Compitrol 888 ATO^®^ and liquid lipids whether (Labrasol^®^ or Labrafil^®^) a clear and homogeneous oil phase was produced when heated to 85 °C with moderate mixing (Kar et al., 2017). The overall amount of solid and liquid lipid inside the blend was 1% (w/v). Double distilled water was used for the aqueous phase (10 mL). As a stabilizer, 1 percent (w/v) SAA whether (Span^®^60 or Brij^®^35) was added. The aqueous phase, as well as lipid phases, was heated at the same temperature for 10 minutes each. The aqueous phase was then poured dropwise to the lipid phase and mixed for 10 minutes at 16,000 rpm using a high-speed magnetic stirrer. After that, the pre-emulsion was treated with a probe sonicator for 10, and 15 minutes (3 minutes, 3 s on/off, and 50% voltage efficiency). The entire volume at the end of preparation was 10 mL. At 4 °C the attained dispersion was stored for further investigations (Duong et al., 2020).

##### Experimental design construction of LORA-NLCs

To investigate the impact of various parameters on the preparation of LORA-NLCs using Design expert^®^ version 13 (Stat Ease, Inc., Minneapolis, MN, USA). The design required building 16 experimental trials corresponding to 2^4^ full factorial design. The following four variables were studied: (X1) solid to liquid lipid ratio, (X2) type of liquid lipid, (X3) type of SAA, and (X4) sonication duration, which were chosen as independent variables, and EE percentage (Y1), PS (Y2) and ZP (Y3), and Q6% (Y4), which were chosen as dependent variables Table 1, the statistical analysis of the factorial design outcomes was performed using Design Expert^®^ (Version 13.0.1, State-Ease Inc., Minneapolis, USA) where Statistical evaluations performed by comparing between various groups of experiments applying the analysis of variants ANOVA considering the null hypothesis (H0). A factor is significant as well as the null hypothesis may be rejected if the P-value is less than 0.05 (Ming et al., 2022).

**Table 1.** 2^4^ Full factorial design for the optimization of LORA-NLCs.

Factors (independent variables) For LORA-NLCs Design	Levels	
A:X1: (solid/liquid lipid) ratio	50:50	20:80
B:X2: liquid lipid type	Labrafil	Labrasol
C:X3: SAA type	Span 60	Birj 35
D:X4: Sonication time (min)	10 min	15 min
Responses (Dependent variables)	Desirability constrains	
Y1: EE %	Maximize	
Y2: PS (nm)	Minimized	
Y3: ZP (mV)	Maximize (absolute value)	
Y4: Q6h (%)	Maximize	

**EE%**; Entrapment Efficiency Percent, **PS**; Particle Size, **ZP**; Zeta Potential; **Q6h**; Amount of drug released after 6 hours, **SAA**; Surface Active Agent, **LORA**; Loratadine, **NLCs**; Nanostructure Lipid Carriers.

#### In-vitro characterization of LORA loaded NLCs

##### Evaluation of entrapment efficiency percentage (EE%)

1 mL of every single formula was centrifuged for one hour at 20,000 rpm and 4 °C employing a cooling centrifuge (Sigma 3 K 30, Germany). After centrifugation, the residue was lysed utilizing methanol and analyzed at 247 nm via a UV-spectrophotometer (Shimadzu UV 1650 Spectrophotometer, Japan) (Abdellatif et al., 2020). The following equation was conducted for the determination of entrapment (Nayak & Tippavajhala, 2021):

(1) EE%=(ED/TD)100

i.e. EE% is the entrapment efficiency percentage, ED entrapped drug concentration and TD is the concentration of the total drug.

##### Evaluation of (PS) particle size and (ZP) zeta potential

PS and ZP were evaluated for LORA-NLCs using the dynamic light scattering (DLS) technique at a 25 °C zeta sizer (Malvern Instruments Ltd., UK). The formulae were appropriately diluted with distilled water in a ratio (1:10) (Gilani et al., 2021a).

##### In-vitro drug release

The in-vitro LORA-NLCs release was assessed using a modified Franz diffusion cell. In simulated ophthalmic conditions, the release rate of LORA-NLCs was investigated, and the percent of drug release after 6 hours (Q6%) was determined. The semi-permeable cellulose membrane (Spectra/Pore dialysis membrane with a 12,000–14,000 Mwt cutoff was soaked in simulated lacrimal fluid SLF solution for 24 hrs at 25 °C with a pH of 7.4 before being used and attached between the donor and receptor compartments. In a donor compartment, 1 mL sample of the prepared LORA-NLCs was inserted (equivalent to 1000 µg of LORA). The media was 20 mL SLF (pH 7.4) applied in the receptor compartment (Mazyed & Abdelaziz, 2020), with continuous stirring at 100 rpm with the magnetic stirrer at 37 °C (Brito Raj et al., 2019). At predefined time intervals, 1 mL sample was collected, and the quantity of LORA released was measured spectrophotometrically at 247 nm using a Shimadzu UV-1601 (Japan) (Li et al., 2021). The samples were taken at 1, 2, 3, 4, 5 as well as 6 hours intervals during *in-vitro* release experiments, sink conditions were maintained in the receptor compartment, and the experiment was repeated three times.

##### Release kinetics

The drug release was processed by applying multiple mathematical models, to investigate both drug-release mechanisms and release kinetics from LORA-NLCs, the Higuchi model, first order, and second-order kinetics were all considered. The model with the greatest coefficient of determination will be used, it was determined (R^2^) (Gurumukhi & Bari, 2021).

##### LORA-NLCs optimization

The desirability function, which allowed for simultaneous analysis of all responses at the same time, was used to determine the best formula. The optimization criteria were chosen to provide a formula with the lowest PS and highest EE percentage, as well as ZP (in an absolute value) and Q6%. The trial with the highest desirability rating was chosen.

##### Compatibility of LORA with the used excipients utilizing FTIR spectroscopy: Fourier transform infrared

FTIR spectroscopy was used to determine interactions between LORA and the excipient. The spectra were recorded for pure LORA, and each pharmaceutical excipients of the optimized formula LORA-NLCs separately, and the optimized formula LORA-NLCs utilizing FTIR 8400 (Shimadzu, Kyoto, Japan). 2–3 mg of every sample was blended with dry potassium bromide and then scanned at the range of 4000–400 cm^−1^ (Karimi Khorrami et al., 2021).

##### Raman spectroscopy

The Raman spectrometer was conducted to evaluate the possible interaction between LORA and other excipients of optimized LORA-NLCs (Farquharson et al., 2021). The Raman spectrum of pure LORA and optimized LORA-NLCs were recorded using (Horiba lab RAM HR evolution visible single spectrometer, Edison, NJ, USA). The 532 nm He-Cd edge laser line with grating 1800 (450–850 nm) and ND filter 0.01% with acquisition time 20 s, accumulations without delay time and spike filter and objective was X0_VIS_LWD. The measurement processes were performed at room temperature in wavelength region 100 and 3199 cm^−1^ (Youssef et al., 2021). Any change or peak shift or broadening was recorded.

##### Transmission electron microscopy (TEM)

The determination of morphology of the optimized formula was throughout utilizing TEM (JEM-1230, Joel, Japan) (Al-mahallawi et al., 2021). Samples were installed on a grid surface of carbon-coated and negatively stained with one percent of the aqueous solution of phosphotungstic acid then dried at room temperature prior visualization (M. Soliman et al., 2022).

#### Study the impact of storage conditions on the optimized formula of LORA-NLCs ocugel

All the optimal NLCs dispersions were kept at 4 °C in addition to 25 °C for one month and samples withdraw and evaluated regarding mean particle size, zeta potential (Zetasizer Nano ZS, Malvern Instruments Ltd., Malvern, UK), EE%, and Q6%. The experiments were conducted in triplicate and all statistical analysis were carried out using GraphPad Instat version 3 software (GraphPad Instat Software, Inc. USA), applying the student’s T-test (Gautam et al., 2022).

#### Fabrication of LORA-NLCs ocugel

LORA-NLCs Ocugel was prepared by utilizing 4% HPMC k100m. The weighed quantity of HPMC k100m was sprinkled during stirring to 1/3 of the requisite quantity of purified water at 80 °C to produce gel, the mixture was stirred for 15 minutes. The total volume was modified to 100 ml by purified water including LORA-NLCs. The gel was left overnight in the refrigerator, LORA-NLCs Ocugel had a final concentration of 1 mg/g LORA (Toma et al., 2022).

#### In-vitro characterization of LORA-NLCs ocugel

##### Physicochemical characters

The visual examination was achieved by applying black and white backgrounds to the visual inspection procedure. The ocugel was noted for appearance, clarity, undesirable foreign particles, as well as turbidity (Bondre et al., 2021). The pH of the ocugel was assessed via a digital pH meter (Elico Pvt. Ltd., India). The pH was determined by dropping the electrode into the gel, all measurements were performed at room temperature (Krambeck et al., 2021).

##### Drug content

Drug content was conducted by diluted of one gram of the LORA-NLCs Ocugel to 100 mL of methanol then stirred for two hours, using a magnetic stirrer. The solution was filtered and analyzed spectrophotometrically at 247 nm (Hajjar et al., 2018).

##### Viscosity

The viscosity of each LORA-NLCs Ocugel formulation was measured at 25 ± 1 °C, by (rotary viscometer, Brookfield Engineering Laboratories, Inc., city, Middleboro, MA, USA), utilizing spindle CP-51 at 50 rpm (Abdelmonem et al., 2020).

##### In-vitro release

The in-vitro release was conducted as mentioned above via utilizing a modified Franz diffusion cell, with similar conditions of NLCs in-vitro release. The samples were filtered through a 0.45-μm filter and evaluated by a UV spectrometer at ƛ_max_ 247 nm (Alshweiat et al., 2020).

#### Ex-vivo studies

##### Ex-vivo trans-corneal penetrability

Ex-vivo trans-corneal penetrability experiment utilizing a modified Franz's diffusion cell. The bovine eyes (gained in the earliest hour of death of a local slaughterhouse) were used for *Ex-vivo* penetration studies. The excised bovine eyes were maintained in 0.9% isotonic sodium chloride and stored at −4 °C, the intact sclera and corneas only were used; the corneas in opaque appearance were discarded (Durgun et al., 2022). Accurately measured 1 g of LORA-NLCs Ocugel, corresponding to 1000 µg LORA, was put in the donor cells. The receptor compartment was loaded with SLF (pH 7.4) at 100 rpm magnetic stirring was carried out at 35 °C. 1 mL of penetration media was removed at the proper time, and the receiver cell was then filled with new media in an equal amount in a certain periods of time 1, 2, 3, 4, 5, 6, 7, 8, 9, and 10 h. The samples were filtered throughout a 0.45 µm membrane and evaluated by utilizing a validated HPLC method (Hassan et al., 2018). The findings were presented as the average of three runs and LORA solution was utilized as a control. An apparent corneal permeability coefficient (cm/h) was calculated using the equation (Eq. (2)) from the quantity of medication that permeated the corneal epithelium as a function of time

(2) $$\text{Papp }=\text{ Jss}/{\text{C}}_{0}$$

where C_0_ is the starting drug concentration (g/cm^2^) and Jss (steady stat flux) indicated the slope of the linear part (g/hr/cm^2^) (Kalam et al., 2022).

##### Confocal laser scanning microscopy study (CLSM)

The confocal image parallel with *Ex-vivo* trans-corneal penetrability was conducted to determine the infiltration of the optimized LORA-NLCs as well as LORA-NLCs Ocugel throughout several layers of cornea, the formulae were fabricated as mentioned before, excepting that LORA was eliminated and 1% (w/v) Fluorescein diacetate (FDA) incorporated in the aqueous phase (Elsayed & Sayed, 2017). Bovine corneas were fixed in diffusion chambers similar to a prior part of the *Ex-vivo* permeation experiment. To simulate the optimized formulae administration in contact with the surface of the eye, FDA-loaded NLCs were installed on the surface of the cornea for 10 hr. The fluorescence in the tissue of the cornea was detected through longitudinal sections, placed in paraffin wax as well as split into several sections utilizing a microtome (Rotary Leica RM2245; Leica Biosystems, Wetzlar, Germany. The inverted microscope (LSM 710; Carl Zeiss, Oberkochen, Germany) was used for the slide’s visualization. The wavelengths of FDA emission and excitation were λ_max_ 516 nm and λ_max_ 497 nm, correspondingly. Confocal figures were provided by LSM Image. Browser software, release 4.2 (Carl Zeiss Microimaging GmbH, Jena, Germany) (Albash et al., 2019). For relative evaluation among the optimized NLCs and NLCs Ocugel, various sections were chosen in corneal tissues in addition to, the light intensity was assessed and analyzed. The test of significance was determined by applying Student’s T-test using GraphPad Instat version 3 software (GraphPad Instat Software, Inc. USA) regarding the significant difference at (*P* < .05). The experiment was repeated for three trials ± SD.

##### Ex-vivo muco-adhesion study

The mucoadhesive force of the fabricated ocular gel was assessed by applying the modified two arms physical balance technique with minor modifications (Cirri et al., 2021). Bovine eyeballs were obtained from a local slaughterhouse and used on the same day of ablation. Two corneas were used; a section of the freshly excised bovine cornea was stuck (using a drop of super glue) to a metal rod connected to the balance whereas the second cornea was stuck on the outside of an inverted beaker placed in a wider beaker containing water not reaching the cornea, acting as a water bath. The temperature was maintained using a hot plate set at 37 °C. A 100 µl formulation sample was added onto the cornea attached to the inverted beaker and the altitude of the second cornea was adjusted so the surfaces of the mucosa of both corneas came into contact. The surfaces of mucosa were held in contact for two minutes prior weights were placed into the pan till the corneas detached. The lowest weight needed to detach both corneas was noted (Eldesouky et al., 2021).

The force of mucoadhesive, represented by the detachment stress (dyne/cm^2^) was calculated by applying the subsequent equation

(3) $$\text{Detachment stress }=\left(\text{dyne}/{\text{cm}}^{\text{2}}\right)=\text{ m }*\text{ g}/\text{A}$$

since m: weight of water represented in gm; g: gravity acceleration taken as 981 cm/s^2^; A: area of contact of bovine’s cornea (cm^2^)

##### In-vivo studies

###### Animals

New Zealand Albino rabbits with healthful eyeballs and without any infections were attained from the House of Animal of Faculty of Pharmacy, Cairo University, Giza, Egypt. The handling and housing of animals were performed according to the guidelines of the Research Ethics Committee (REC) of Cairo University. The protocol of research was recognized by the Animal Care Committee of the National Research Center (Cairo, Egypt) and was accepted by the Ethics Committee (PI 2606 in February 2020).

###### Ocular irritation study (Draize test)

In vivo monitoring of ocular irritation was determined by classical Draize test of optimized LORA-NLCs Ocugel. Four adults, white New Zealand male albino rabbits each one balancing 2–3 kg, were stable and had no abnormalities. The standard conditions such as moisture, air, temperature, and light, in addition to food, and water were maintained. The formula was installed in the left eye particular in the conjunctival sac, as well as the right eye was reserved as control by applying saline at intervals 0, 1, 2, 3, 4, 5, 6, 24, 48, and finally at 72 hours then 7, 14, and 21 days after installation, each eye of rabbit was examined to assess the irritation grade. Draize test applies a scoring system varying from 0 (no irritation) to 3 (maximum redness and irritation) in the iris, cornea, conjunctiva, and pupil, in addition to the anterior chamber. At the end of the experiment eyeballs of the rabbits instantaneously were taken out for further histopathological studies (El-Emam et al., 2021).

###### Histological examination

Following the Draize test, the histo-pathological examination was done to detect the safety of LORA-NLCs Ocugel on ocular tissues, the rabbits were sacrificed by utilizing phenobarbital sodium injection in marginal vein. Tissue specimens were rinsed and dehydrated in high grades of alcohol after being trimmed off. The specimens were inserted in paraffin blocks after being cleared in xylene and sectioned with a thickness of 4–6 µm. for histopathological examination via the electric light microscope. The tissue sections were deparaffinized utilizing xylol and stained using hematoxylin and eosin (H&E) (Suvarna et al., 2018).

###### Pharmacokinetic parameters study

The rabbits were separated into the following two groups at random (3 rabbits on each); the first one was treated with LORA-NLCs Ocugel, and the second was treated with the pure LORA loaded in HPMC-K100 gel. During the procedure, Sodium pentobarbital (30 mg/kg) was administered into the marginal ear vein to keep the rabbits under anesthesia during the experiment. Following administration of 100 µL of each preparation, 100 µL aqueous humor was withdrawn through a one-milliliter insulin needle at 1, 2, 4, 6, 8, 10, and 24 hours as well as placed in a centrifuge tube. The Precipitated protein was obtained by vortex and mixing with methanol (0.5 mL). Precipitated protein was separated through centrifugation for 10 min at 10,000 rpm, subsequently, the LORA concentration was determined in the supernatant using HPLC, the analysis detection was performed on a C18, where the optimal conditions were presented as the mobile phase contained a 50 mM solution of acetate buffer (adjusted with glacial acetic acid at pH = 3)/methanol (15/85, v/v) as well as the flow rate was 1 mL/min. Where the volume of injection was 20 µL. Additionally, the temperature was kept at 25 °C during the experiment. The identification and detection of LORA were performed at 248 nm (Spac et al., 2016). The parameters of pharmacokinetics were computed by the noncompartment method using the WinNonlin software of pharmacokinetics (Certara Inc., Princeton, NJ, USA). All pharmacokinetics parameters of different formulae were compared statistically using GraphPad Instat version 3 software (GraphPad Instat Software, Inc. USA), applying the student’s T-test (Abdelmonem et al., 2021).

## Statistical analysis

The experiments were conducted in triplicate, all values, and their mean of three measurements with standard deviation (±SD) was recorded. The statistical analysis was carried out using GraphPad Instat version 3 software (GraphPad Instat Software, Inc. USA). The disparities between various groups of the experiment were computed by applying the analysis of variants ANOVA. On the other hand, student’s T-tests were used to compare two groups considered (P-values < .05) significant difference.

## Results and discussion

### Statistical analysis model fitting

The goal of the process of optimization is to figure out the levels of variables that are essential for the development of products with high quality. Design of experiments-DOE is one of strategy for planning tests and analyzing the results to determine which factors, from several variables are the most essential contributors to any process. The approach allows conducting a small number of experiments in which a variety of independent factors can be changed to investigate their impact on various responses (Tavares Luiz et al., 2021).

The prepared LORA-NLCs were optimized by employing full factorial design applying Design-expert^®^ software that established 16 experimental trials as shown in Table 2. Four independent variables were studied: the: (X1) solid to liquid lipid ratio, (X2) type of liquid lipid, (X3) type of SAA, and (X4) sonication duration, which was selected as independent variables, and EE percentage (Y1), PS (Y2) and Zp (Y3), and Q6% (Y4). Table 3 showed that the value of adjusted R^2^ and predicted R^2^ of various responses were in acceptable agreement since the difference between each of them was fewer than 0.2. Moreover, adequate precision was noticed to be higher than the desired value (4) for Y1, Y2, Y3, and Y4. Therefore, this model could be suitable for the design space navigation (Badr-eldin et al., 2022), whereas the fitted model was an Analysis of variance table partial sum of squares—type III for all responses.

**Table 2.** Output data of the 2^4^ full factorial analysis of NLCs formulations.

NLCs formulation	X1	X2	X3	X4	Y1	Y2	Y3	Y4
	(Solid/liquid lipid) ratio (% w/w)	Liquid lipid type	SAA type	Sonication Time (min)	EE %	PS (nm)	ZP (mV)	Q6h (%)
LORA-NLCs-1	50/50	Labrafil	Span 60	10	91.29 ± 0.52	100.90 ± 0.65	−31.21 ± 0.42	58.16 ± 1.82
LORA-NLCs-2	50/50	Labrasol	Span 60	10	95.78 ± 0.67	156.11 ± 0.54	−40.10 ± 0.55	99.67 ± 1.09
LORA-NLCs-3	50/50	Labrafil	Brij 35	10	70.6 ± 0.98	187.93 ± 0.69	−22.42 ± 0.18	53.55 ± 2.07
LORA-NLCs-4	50/50	Labrasol	Brij 35	10	79.21 ± 0.28	253.41 ± 1.07	−30.82 ± 0.25	97.92 ± 1.48
LORA-NLCs-5	20/80	Labrasol	Span 60	10	92.85 ± 0.31	250.92 ± 1.50	−38.78 ± 0.47	92.55 ± 1.65
LORA-NLCs-6	20/80	Labrafil	Span 60	10	92.32 ± 0.54	198.19 ± 0.76	−30.16 ± 0.29	58.01 ± 1.87
LORA-NLCs-7	20/80	Labrasol	Brij 35	10	76.79 ± 1.09	317.11 ± 2.85	−28.54 ± 0.15	85.56 ± 2.84
LORA-NLCs-8	20/80	Labrafil	Brij 35	10	70.51 ± 0.84	247.32 ± 1.06	−20.27 ± 0.23	68.14 ± 1.06
LORA-NLCs-9	50/50	Labrafil	Span 60	15	91.84 ± 0.32	376.11 ± 2.72	−18.18 ± 0.12	87.98 ± 2.73
LORA-NLCs-10	50/50	Labrasol	Span 60	15	96.11 ± 0.73	388.81 ± 2.43	−23.43 ± 0.34	98.43 ± 1.85
LORA-NLCs-11	50/50	Labrafil	Brij 35	15	71.21 ± 0.54	351.52 ± 2.72	−10.41 ± 0.14	87.56 ± 2.06
LORA-NLCs-12	50/50	Labrasol	Brij 35	15	77.87 ± 0.30	435.61 ± 3.29	−15.28 ± 0.11	81.89 ± 1.37
LORA-NLCs-13	20/80	Labrasol	Span 60	15	90.74 ± 0.64	588.40 ± 4.51	−18.45 ± 0.25	75.77 ± 1.72
LORA-NLCs-14	20/80	Labrafil	Span 60	15	87.44 ± 1.07	456.18 ± 3.27	−14.62 ± 0.13	94.33 ± 1.07
LORA-NLCs-15	20/80	Labrasol	Brij 35	15	85.53 ± 0.76	467.71 ± 3.63	−11.21 ± 0.15	97.55 ± 2.67
LORA-NLCs-16	20/80	Labrafil	Brij 35	15	81.82 ± 0.32	404.11 ± 3.28	−7.54 ± 0.07	51.06 ± 1.95

Note: Data represented as mean ± SD (*n* = 3). Abbreviations: **EE%**; Entrapment Efficiency Percent, **PS**; Particle Size, **ZP**; Zeta Potential; **Q6h**; Amount of drug released after 6 hours, **SAA**; Surface Active Agent, **LORA**; Loratadine, **NLCs**; Nanostructure Lipid Carriers.

**Table 3.** Experimental runs, independent variables, and measured response of the 2^4^ full factorial experimental design of LORA-NLCs.

Responses	EE %	PS (nm)	ZP (mV)	Q6h (%)
Adequate precision	22.33	30.79	32.89	39.67
Adjusted R^2^	0.98	0.99	0.98	0.99
Predicted R^2^	0.97	0.98	0.96	0.97
Significant factors	X2.X3.X4	X1.X2.X3.X4	X2.X3.X4	X2
Predicted value of optimum formula (NLC2)	94.56	153.78	37.65	98.23
Observed value of optimized formula (LORA-NLC-2)	95.78	156.11	40.10	99.67
Model fitting	Analysis of variance table partial sum of squares—type III for all responses.			

**EE%**; Entrapment Efficiency Percent, **PS**; Particle Size, **ZP**; Zeta Potential; **Q6h**; Amount of drug released after 6 hours, **LORA**; Loratadine, **NLCs**; Nanostructure Lipid Carriers.

#### Impact of formulation variables on the EE%

**Figure 1.**
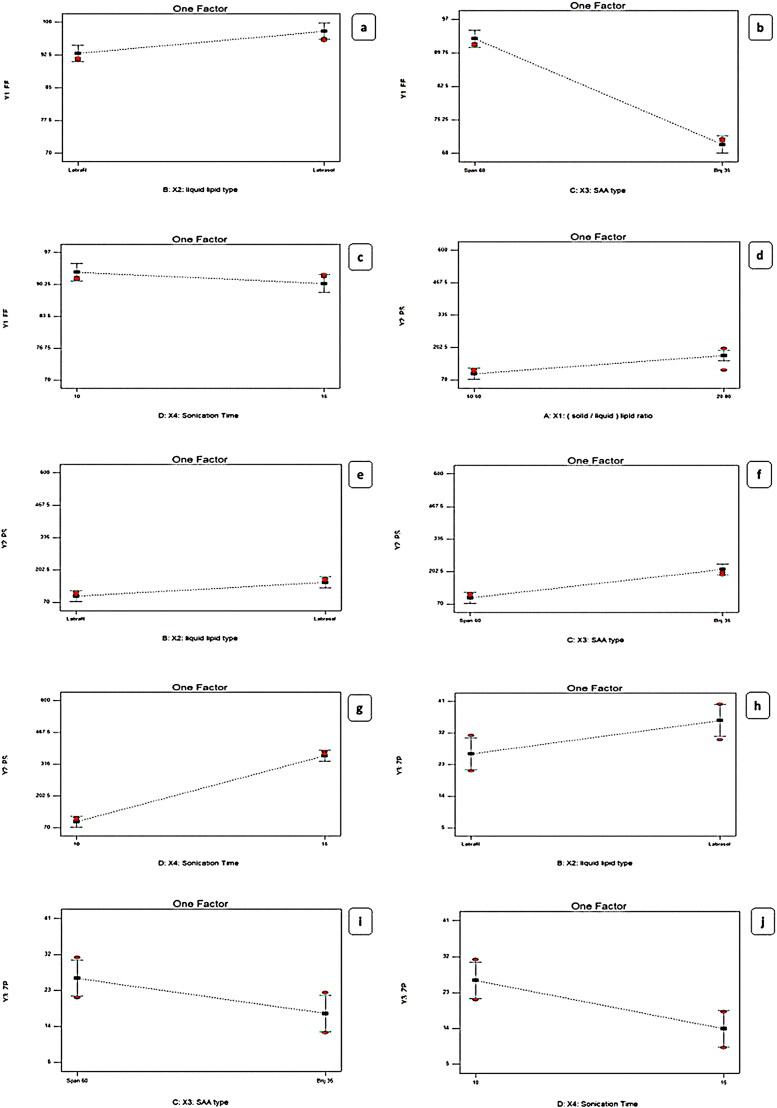
Linear plot of the effect of formulation variable on EE% (a–c), PS (d–g), and ZP (h–j). EE%; Entrapment Efficiency Percent, PS; Particle Size, ZP; Zeta Potential; SAA; Surface Active Agent, LORA; Loratadine, NLCs; Nanostructure Lipid Carriers.

EE% ranged from (70.51 ± 0.84 to 96.11 ± 0.73) %, as shown in Figure 1 and Table 2, for the significant effect of independent variable X2: liquid lipid type, X3: SAA type, and X4: Sonication Time (min). The type of liquid lipid effect on EE%, where both Labrafil^®^ and Labrasol^®^ have two different fundamental roles impacted EE%. The first is that, both were considered PEGylated lipid and solubility enhancers for the hydrophobic drug, which augments the amount of drug incorporated into the nanostructured (Houacine et al., 2020) since LORA exhibited as a model of ‘very lipophilic’ drug (Sarheed et al., 2020). However, the higher EE% of LORA-NLCs fabricated with Labrasol^®^ than Labrafil^®^, might be due to various crystal defects in the solid lipid as well as imperfections in the highly ordered crystal matrix caused by Labrasol^®^, this result complies with the previously reported (Ammar et al., 2021), regarding the effect of incorporation of Labrasol^®^ into the solid lipid matrix rendering the NLCs to become more imperfect, thus yielding, sufficient space in solid lipid matrix for a large quantity of drug to lodge effectively.

Furthermore, the EE% results declared that the X3: Type of SAA had a significant (*p* < .0001) effect indicating that the entrapment efficiency was augmented with a lowering in the HLB value of the surfactant. Span^®^ 60 produced the highest EE% of the hydrophobic drug which can be attributed to the presence of the long-saturated alkyl chain (C18) of Span^®^ 60, responsible for its high lipophilicity with low (HLB values 4.7) (Abdel-Aziz et al., 2021), while Brij^®^35 (polyoxyethylene alkyl ethers) was surfactant that differs in the quantity of hydrophilic moiety having short alkyl chain length (C12) with high (HLB value of 16.9) and high hydrophilic properties (Sayed et al., 2020).

Concerning the impact of time of sonication X4, it was concluded that by reducing the time of sonication from 15 min to 10 min the EE% significantly amplified, possibly because of decreasing the leakage of LORA to the exterior aqueous medium throughout disruption then nanovesicles re-aggregation instead of encapsulation (Zaki et al., 2022). On the other hand, the effect of X1: solid to liquid lipid ratio on EE% of LORA-NLCs, displayed a non-significant impact (*P* > 0.05).

#### Impact of formulation variables on PS

The particle size of the fabricated LORA-NLCs fluctuated from (100.90 ± 0.65 to 588.40 ± 4.51) nm as presented in Table 2. Considering X1: solid to liquid lipid ratio, the ratio (50:50%) had significantly (*P* < .0001) decreased in PS compared with (20:80%), this result can be attributed to liquid lipid being excluded during the formation of particle. once the system was cooled, solid lipids within the solidification process at a low temperature rapidly solidify resulting from their owning high melting point and then started to be arranged as nanoparticles, whereas liquid lipids owning soft structures that might stay outside or distributed randomly (Madan et al., 2020). Furthermore, (Patel et al., 2021) reported that when solid-lipids were increased, a reduction in the size of NLCs was shown in Figure 1. Moreover, the variation of the type of liquid lipid X2 was found to show a significant effect on PS (*P* < .0001) the PS increased with Labrasol^®^ compared with Labrafil^®^, this may be because of PEG moieties of both liquid lipids where Labrafil^®^ with 6 PEG repeating units produced PS smaller than Labrasol^®^ having 8 PEG that might be extended at the surface of particle producing an enlarging in PS (Safwat et al., 2017).

Regarding the effect of the X3: type of SAA impact on PS, a decrease in HLB value resulted in smaller PS, this result is inconsistent with (Rubab et al., 2021), this could be attributed to the correlation of NLCs size with surfactant hydrophobicity, where on decreasing the surface energy with increasing the SAA, hydrophobicity occurs. Nevertheless, the enlarged PS was obtained with higher HLB surfactant as their presence in the hydrophilic layer encourages water absorption (Sayed et al., 2020). The PS of LORA-NLCs with Span^®^60 were significantly (*P* = .0142) lower than those for Brij^®^35, this can be probably because of the smaller value of HLB of Span^®^60 (HLB = 4.7, C18), compared with a polyoxyethylene alkyl ether surfactant with (HLB = 16.9, C12) due to its prominent hydrophilic nature.

X4: sonication time positively impacted PS (<.0001), where the increase of sonication time resulted in larger PS. This finding revealed that at high sonication time, the high surface area of the highly reduced PS creates surface charges inducing interactive forces which in turn led to further agglomeration and PS increment as reported by (Radwan et al., 2020). Thus, the optimum time of sonication was 10 min to achieve the required particle size (Walunj et al., 2020). These outcomes agreed with data of EE%, where a higher entrapment of LORA in NLCs was attained upon utilizing Labrasol^®^

#### Impact of formulation variable on ZP

The formulated LORA-NLCs were found to exhibit negative ZP values ranging from (−7.54 ± 0.07 to −40.10 ± 0.55) mV as revealed in Table 2. It is worthy to mention, as previously reported, that the ZP value above −30 mv indicates good physical stability of the dispersed system against aggregation through electrostatic repulsion (Elfadl et al., 2021). The ANOVA results showed that the independent variable X2: liquid lipid type (*P* = .0038) Figure 1, had a significant effect on ZP this might be due to the PEG chains of both Labrafil^®^ (6 PEG units) and Labrasol^®^ (8 PEG units) that could cover the surface of particles reducing their charge density. The presence of Labrasol^®^ relatively, with a longer chain of PEG that coved the surfaces of particles, was noticed to enhance zeta potential which is frequently the important factor in realizing how the process of the aggregation and dispersion is applied (Ghanem et al., 2021)

Moreover, X3: SAA type (*P* = .0005) was also found to have a significant influence on ZP. The type of SAA plays a crucial role in the physical stability of LORA-NLCs. The formulations exhibited a high negative zeta potential for Span^®^60, which could be referred to as increasing OH − ion concentration producing highly negative charges which gives the vesicles higher stability contrasted to that gained by Brij^®^35. This could be also attributed to the higher hydrophilicity nature of Brij^®^35 having fewer lipophilic repeating units (m = 12) with (HLB 16.9) compared with Span^®^ 60 (HLB 4.7) which might provide a shield of negative charge on vesicular bilayer’s surface (Abdelbari et al., 2021).

Subsequently, this has resulted in a lower ZP value significantly, the same result was reported by (Mosallam et al., 2021). Additionally, sonication Time (min) (*P* < .0001) significantly affected zeta potential values. Obviously, the time of sonication had impacted negatively the values of zeta potential, where increasing the sonication time led to a decrease in the ZP values and hence, a decrease in the stability of the dispersion system (Zaki et al., 2022). Alternatively, the X1: solid to liquid lipid ratio was found to show no significant effect on ZP (*P* = .2711).

#### Impact of formulation variable on in-vitro release

**Figure 2.**
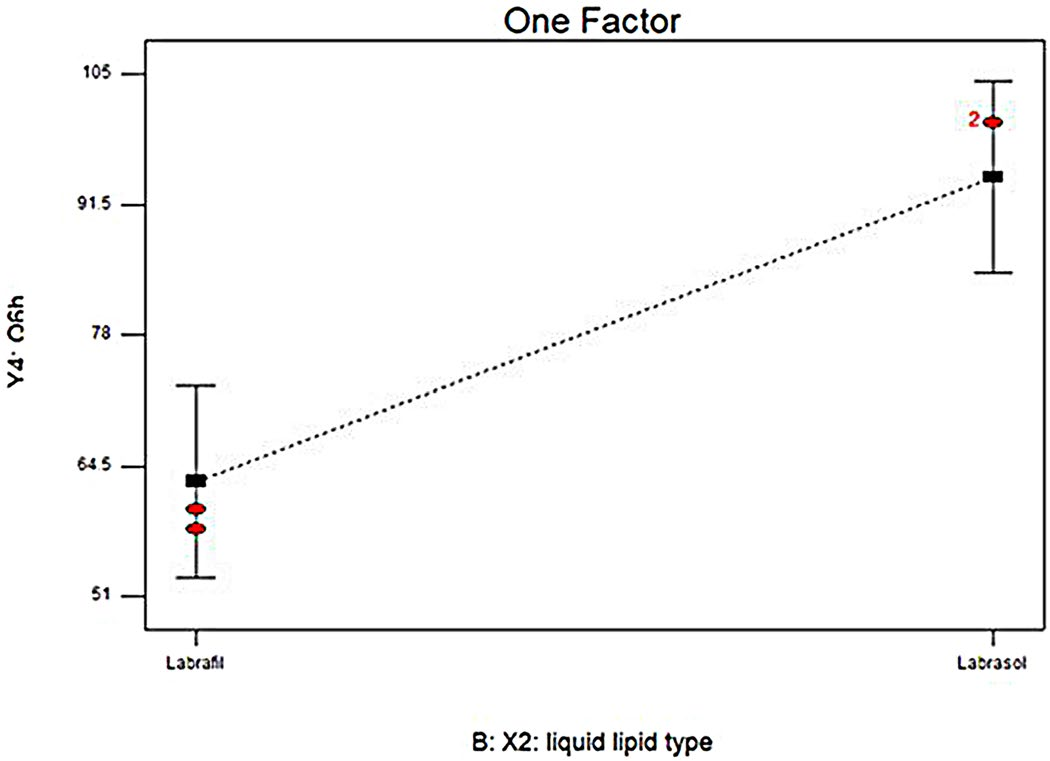
Linear plots for the effect of (X2) type of liquid lipid on Q6h. Q6h; Amount of drug released after 6 hours.

To get insights into the impact of formulation variable on the LORA-NLCs *in-vitro* release, results obtained from ANOVA indicated that liquid lipid type (X2) had a significant (*P* < .0001) impact on Q6%, Table 2. NLCs belonging to Labrasol^®^ showed higher LORA released as compared to those belonged to Labrafil^®^ Figure 2. This finding could be related to the previously mentioned justification, that the incorporation process of liquid lipids into the solid lipid matrix produced more spaces in the solid lipid matrix, hence, enhancing the release of the loaded drug. Additionally, the higher HLB value of Labrasol^®^ (HLB = 12) might have increased the solubility of the hydrophobic drug in the aqueous phase and subsequently enhanced its release (Sweed et al., 2021).

#### Release kinetics

The drug release from LORA-NLCs formulae was fitted in several mathematical models to identify the mechanism of release. The values of the regression coefficient imply that the Higuchi model was the best fit for the LORA-NLCs release profile (Ma et al., 2022).

#### Selection of the optimized LORA-NLCs

Certain requirements were defined in Design Expert^®^ software version 13 to choose the optimum equations. The lipid nanovesicles with the highest Entrapment efficiency percent, Zeta potential (in the absolute value), Q6%, and lowest PS were selected at optimum conditions. The formula was prepared using Compritol 888^®^: Labrasol^®^ ratio of 50:50%, with Labrasol^®^ as a type of liquid lipid, and Span^®^ 60 as a surfactant, with a sonication time of 10 min was the optimized formula LORA-NLCs-2 which satisfied these requirements and accomplished the highest desirability value (0.884).

This formula showed EE% of 95.78 ± 0.67%, PS of 156.11 ± 0.54 nm, ZP of −40.10 ± 0.55 Mv, and Qh6% of 99.67 ± 1.09%. Particularly, to validate the experiment, the observed and predicted responses of LORA-NLCs-2 were compared as prior displayed in Table 3. A high correlation was noted between the values of observed and predicted responses. Accordingly, the optimized LORA-NLCs-2 was considered promising to be used in further investigation.

#### Fourier transform infrared (FTIR) spectroscopy

**Figure 3.**
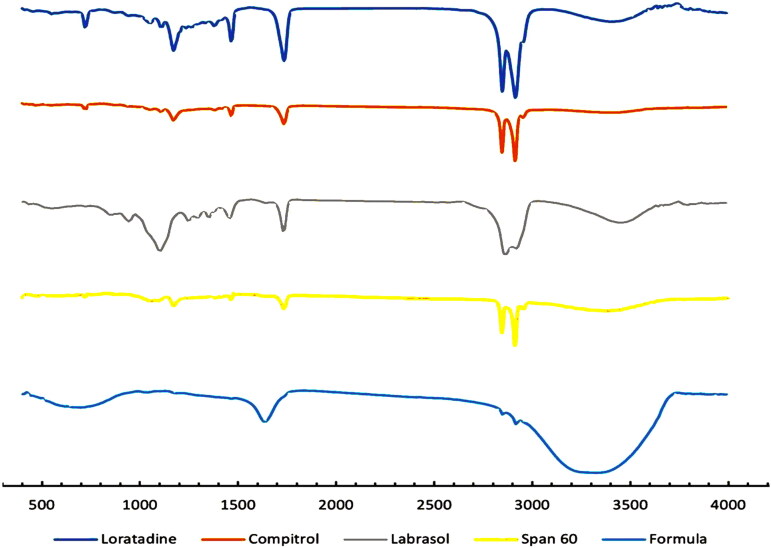
FTIR spectra of pure Loratadine (LORA) and each pharmaceutical excipients of the optimized formula LORA-NLCs separately, and the optimized formula LORA-NLCs.

FTIR spectroscopy of pure LORA, optimized LORA-NLC-2, and all incorporated excipients were displayed in Figure 3. Prominent pure LORA peaks in 1701, 1643, 1440, 1228, 1095, and 999 cm^−1^, corresponding to the C = O, C = N, C = C, C–N, C–Cl, and = C–H vibrations, respectively which were also observed in optimized LORA-NLCs-2 spectra. This suggests no possible interaction between LORA and excipients and confirms the stability of prepared LORA-NLCs (Emami et al., 2020).

#### Raman spectroscopy

**Figure 4.**
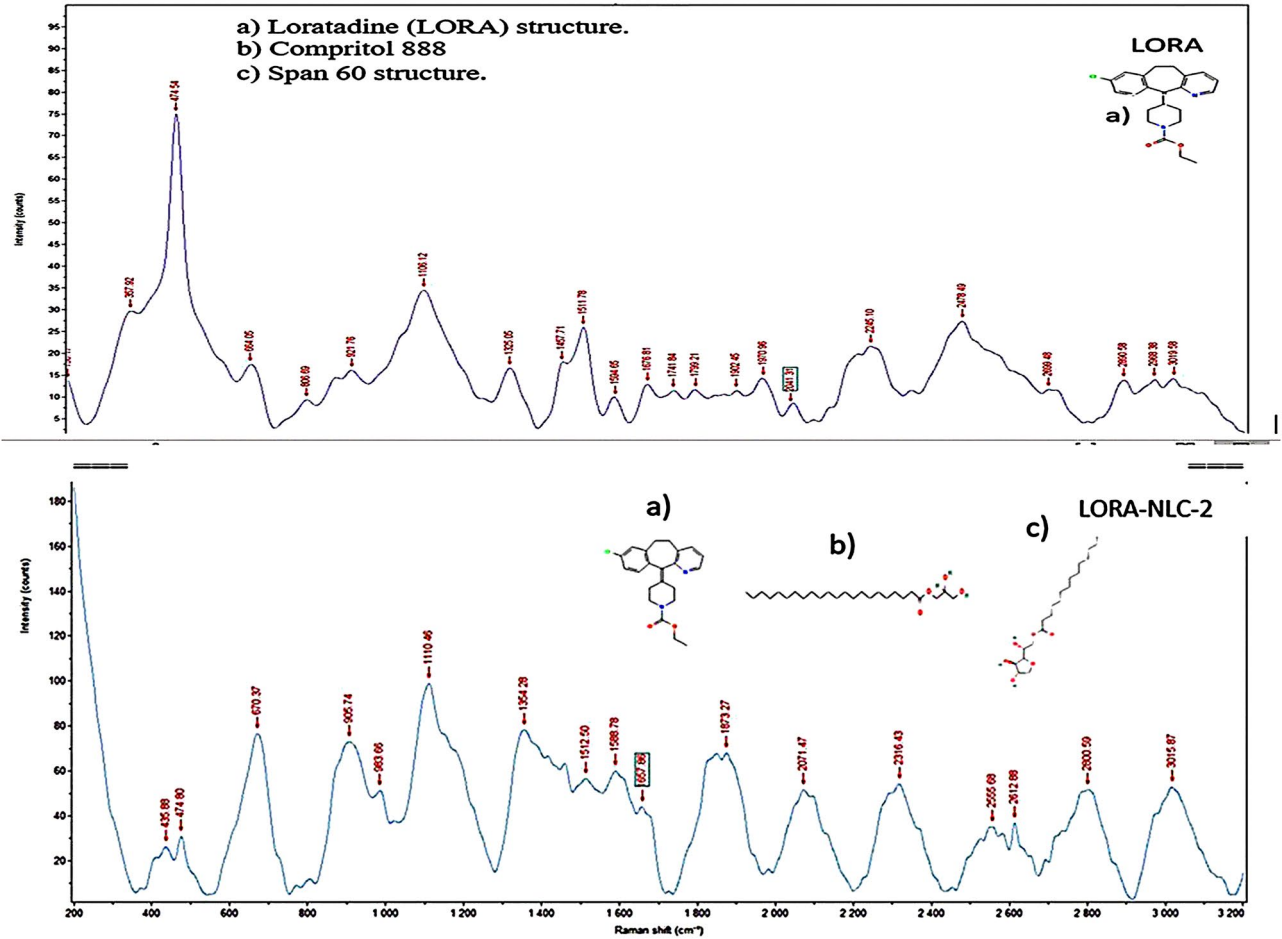
Raman spectra of pure Loratadine powder (LORA) and optimized formula (LORA-NLCs-2).

Raman spectroscopy of optimized LORA-NLCs-2 as depicted in Figure 4, displayed no drastic change or peak shift or broadening of the most distinct peaks of LORA at 1741.8 cm^−1^ for C = O, 664.05 cm^−1^ for C-Cl, and 1630 cm^−1^ for C = N and excipients, subsequently Raman spectroscopy showed no chemical interaction among the excipients and LORA.

#### Transmission electron microscopy (TEM)

**Figure 5.**
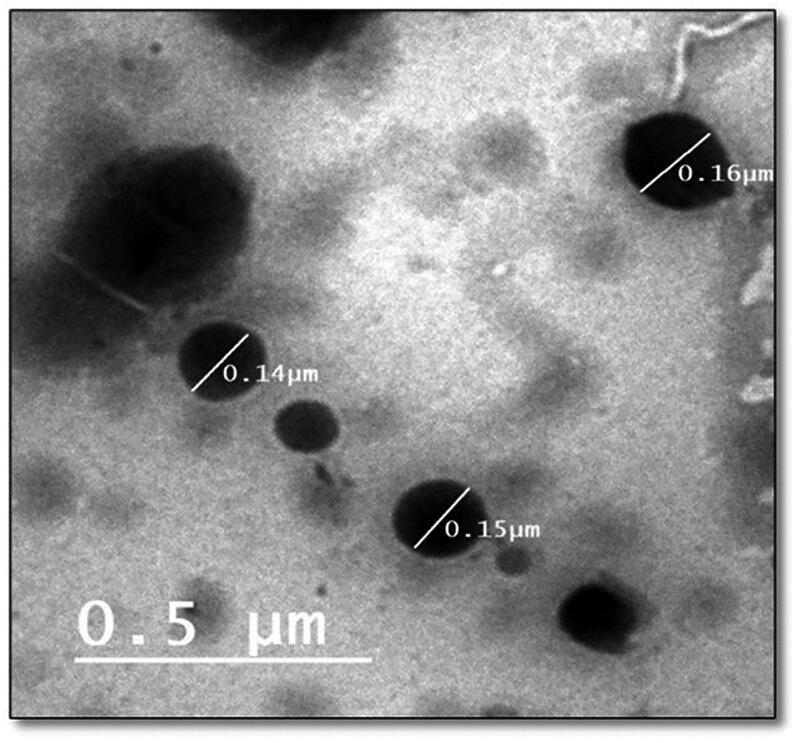
Transmission electron micrograph of the optimized LORA-NLCs-2.

The external morphology of the optimized LORA-NLCs-2 formula was determined utilizing TEM analysis. As shown in Figure 5. the morphological analysis displayed that the nanoparticles had a uniform size distribution and spherical shape. Accordingly, the average size of particle obtained by Zetasizer well agrees with TEM results.

#### Impact of storage condition on the optimized formula of LORA-NLCs ocugel

The statistical analysis T-test showed a significant difference (*P* < .05) of EE% observed at 25° and 4 °C compared with freshly prepared one this might be due to recrystallization of solid lipid content in NLCs optimized formula which led to drug leakage with a subsequent decrease in EE% (Osanlou et al., 2022). Besides that, a reduction of ZP with time (*P* < .05) was also detected for all storage temperatures. This reduction can be explained by the increase the kinetic energy which can lead to changes in the crystalline structure of the lipid (Azevedo et al., 2021), subsequently, a significant change (*P* < .05) was detected in PS this might be due to the effect aggregation of particles. On the other hand, the results of the Q6h (%) evaluation indicated there was no significant change (*P* > 0.05). Data in Table 4 showed the percent drug retention data indicated the effect of temperature on the stability of NLCs. The lower loss in percent drug retention suggests that the NLCs were more stable when stored at 4 °C, where visual inspection displayed no phase separation and gel formation have been observed. These outcomes reveal the LORA-NLCs-2 acquired good stability at 4 °C

**Table 4.** Impact of storage conditions on the optimized formula of LORA-NLCs ocugel.

Parameters	LORA-NLCs-2 freshly prepared	LORA-NLCs-2 after one month of storage at 4 °C	LORA-NLCs-2 after one month of storage at 25 °C
EE%	95.78 ± 0.67	91.08 ± 1.18	90.71 ± 2.07
Ps (nm)	156.11 ± 0.54	166.00 ± 1.84	162.83 ± 1.37
ZP (mV)	−40.10 ± 0.55	−37.22 ± 0.62	−32.87 ± 1.63
Q6h (%)	99.67 ± 1.09	98.19 ± 1.09	98.79 ± 2.74

Abbreviations: **EE%**; Entrapment Efficiency Percent, **PS**; Particle Size, **ZP**; Zeta Potential; **Q6h**; Amount of drug released after 6 hours, **LORA**; Loratadine, **NLCs**; Nanostructure Lipid Carriers.

#### Evaluation of LORA-NLCs ocugel

The physicochemical character of LORA-NLCs Ocugel was found to be clear upon observation without any phase separation (Gilani et al., 2022). The pH of LORA-NLCs Ocugel was appropriate to ocular pH (7.11 ± 0.52) which would aid the penetration of LORA-NLCs-2 into several layers of the cornea with no irritation. The content of the drug in NLC-based gel was determined to be in the adequate range of (98.62% ± 1.31%) displaying the uniformity of content of the drug in the gel matrix (Wairkar et al., 2022). Regarding the viscosity, among the studied gel, it was found to be 2736 cp which might retard NLCs diffusion through the gel and sustain its release and extend the residence time upon the ocular route (Fathalla et al., 2015), this finding agree also with (Kumar et al., 2019), whereas in the reported study, the LORA-NLCs-2 release was retarded up to 12 h, with Q12% (90.49 ± 1.32%) by incorporating the LORA-NLCs-2 to HPMC K 100, considered as a release retarded polymer and viscosity enhancer (Vooturi et al., 2020).

### Ex-vivo trans-corneal penetrability study

**Figure 6.**
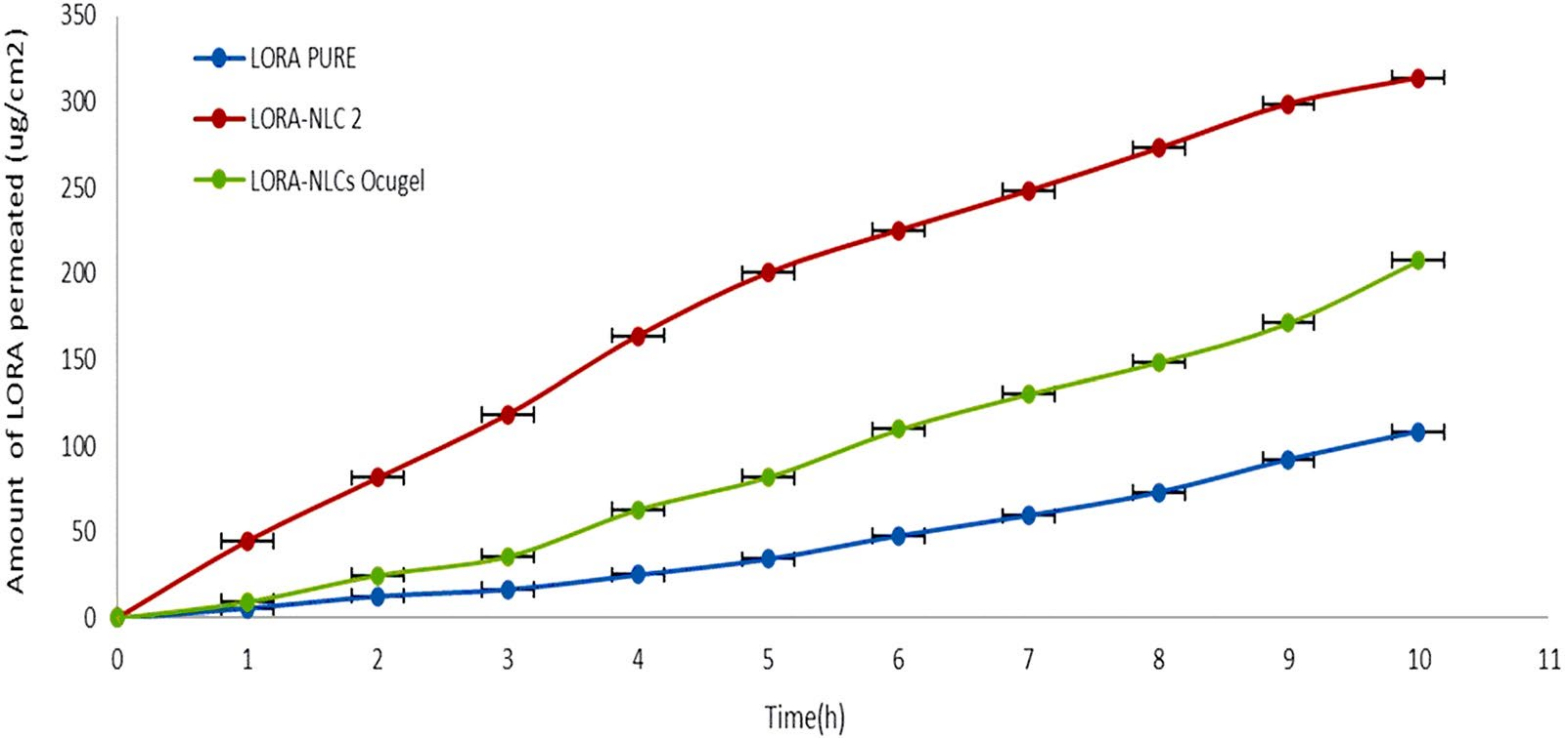
Cumulative amount of LORA permeated per unit area across excised bovine cornea via NLCs and NLCs Ocugel relative to LORA aqueous solution. Data were illustrated as mean ± S.D; n = 3.

Figure 6 represents the cumulative variation of the amount of LORA penetrated through the excised bovine cornea of NLCs and NLCs Ocugel compared with LORA aqueous drug solution, during 10 h. It is obvious that there was a significant (*P* < .05) increase in the trans-corneal penetrability of LORA from LORA-NLCs, and LORA-NLCs Ocugel compared with the aqueous drug dispersion, where NLCs provided their ability to solubilize, enhance the encapsulation and penetration efficiency of hydrophobic compound (Tang et al., 2022b). Meanwhile, Labrasol^®^ has excellent solubilization and penetration-enhancing effect which promoted the permeability of LORA-loaded NLCs through the corneal layer (Tasharrofi et al., 2022).

Alternatively, LORA-NLCs Ocugel had shown an approximately two-fold increase in *Ex-vivo* trans-corneal penetrability and sustained retention capacity of the drug on the surface of the cornea compared with LORA dispersion. LORA-NLCs showed greater Jss and ER relative to LORA-NLCs Ocugel after 10 h as indicated in Table 5. This result reveals that the prepared ocugel, using HPMC K100 polymer, increased the viscosity that subsequently, may increase the retention time thus, augment the contact time of LORA-NLCs to the surface of the cornea against the physiological process as blinking, reflux, and tear drainage (Iqbal et al., 2012).

**Table 5.** Corneal penetrability parameters of LORA after application of LORA dispersion, LORA-NLCs, and LORA-NLCs ocugel.

Corneal permeability parameters	LORA aqueous dispersion	LORA-NLCs	LORA-NLCs ocugel
Total amount of LORA permeated per unit area after 10 h (μg/cm^2^)	108.21 ± 1.78	313.88 ± 2.76	207.73 ± 1.93
J_max_ (μg/cm^2^/h)	10.68 ± 0.54	31.64 ± 0.87	20.84 ± 0.62
ER	1	2.96	1.95

### Confocal laser scanning microscopy (CLSM)

**Figure 7.**
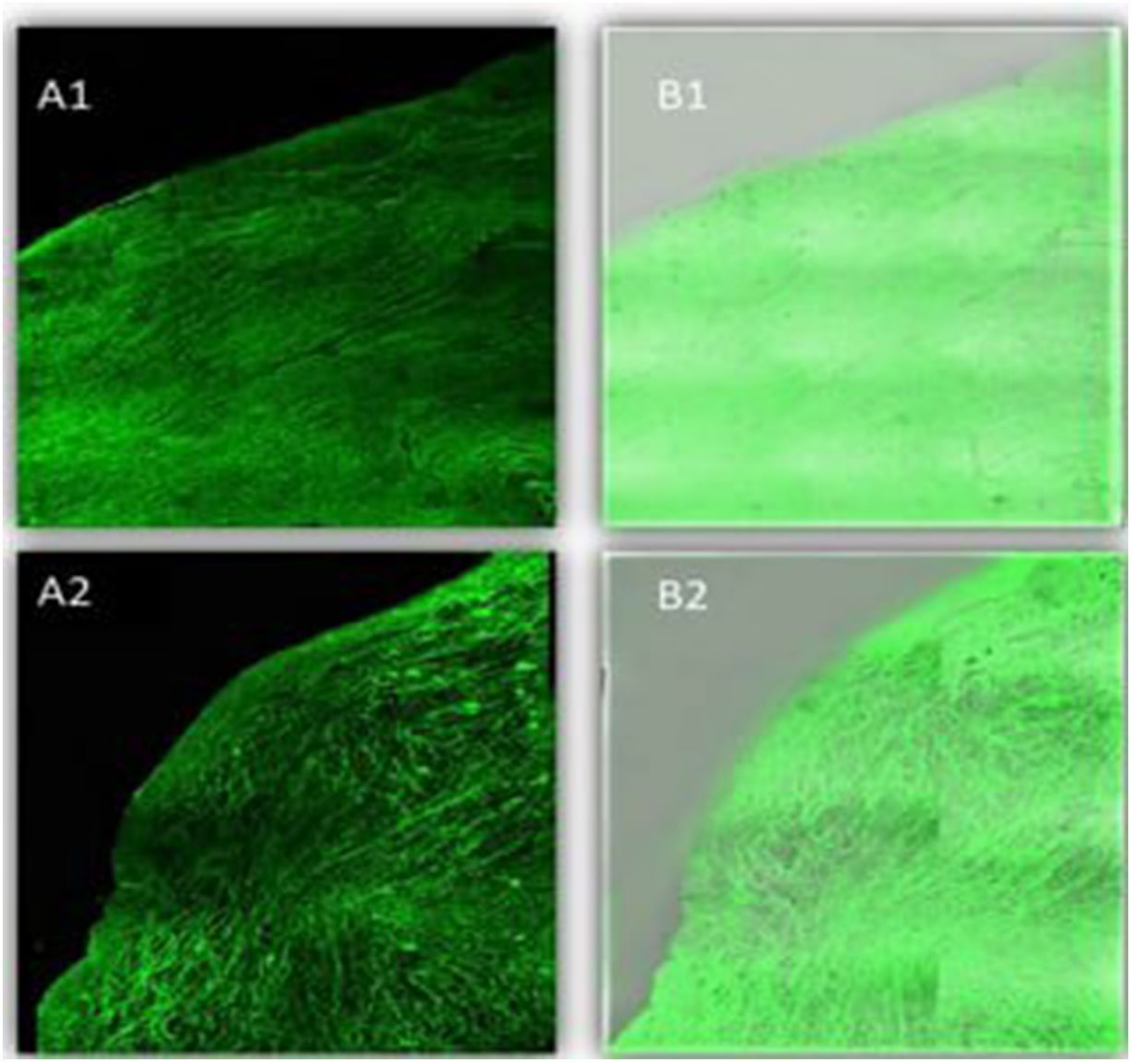
A tile scan confocal laser microscope photomicrograph of a longitudinal section in bovine cornea treated with FDA-loaded NLCs, and NLCs Ocugel. Note: (1) NLCs ocugel, (2) optimized NLCs, (A) Fluorescence light, and (B) merge between fluorescence light and transmitted light. Abbreviations: FDA, fluorescein diacetate; NLCs, nanostructure lipid carriers.

CLSM manifested the penetration potency across the corneal layer by utilizing FDA that incorporated selected NLCs and NLCs Ocugel instead of LORA, where the intensity of fluorescent light, after the ocular delivery was determined, Figure 7. Firstly, scans of the longitudinal section were taken and showed the depth of penetration of nano lipid carrier in the tissues of the cornea, both preparations displayed deep penetration across the layers of the cornea, as demonstrated by high and great fluorescence intensity and homogeneous distribution. This deep penetration of NLCs might be ascribed to the small nanoparticle size, which provided a high surface area. In addition, the presence of Labrasol^®^ liquid lipid enhanced drug penetration. Nevertheless, by computing the maximum fluorescent light intensity, moreover, there was (*P* > 0.5) non-significant difference among the NLCs and NLCs Ocugel as the average intensity was 153.639 ± 1.67 and 152.627 ± 1.08 for NLCs and LORA-NLCs, Ocugel, respectively, thus it is worth to note that the fabricated ocugel utilizing HPMC K100 polymer had no impact on penetration on NLCs through the corneal layers.

### Ex-vivo muco-adhesion test

Muco-adhesion, expressed as the force required to detach the formulation from the corneal surface of LORA-NLCs Ocugel utilizing HPMC K100 polymer was generated by applying the formulation between two face-to-face placed bovine corneas. The value recorded for LORA-NLCs Ocugel was 22,305.5 ± 247.3 dyne/cm^2^ this can be correlated to the ability of HPMC K100 polymers, with its hydrophilic functional groups such as hydroxyl groups, to form electrostatic and hydrophobic interactions, as well as, hydrogen bonding with cornea surface (Dave et al., 2021). Thus, LORA-NLCs Ocugel showed good muco-adhesion force for attaining prolonged residence in the lachrymal fluid by increasing the contact time of the ocugel on the corneal surface.

### In-vivo studies

#### Ocular irritation study (Draize test)

An ocular irritancy test was performed after 21 days and revealed a lack of any inflammatory-related symptoms, such as redness, edema, or increased tear production. Evaluating the score of ocular irritation was performed following administration with zero scoring at specific intervals, and the ocular examination showed clear and flat cornea, normal conjunctiva and iris, regular and reactive pupil, and formed clear anterior chamber. Therefore, these results verify the safety of optimized LORA-NLCs Ocugel for topical application to the eye.

#### Histological examination

**Figure 8.**
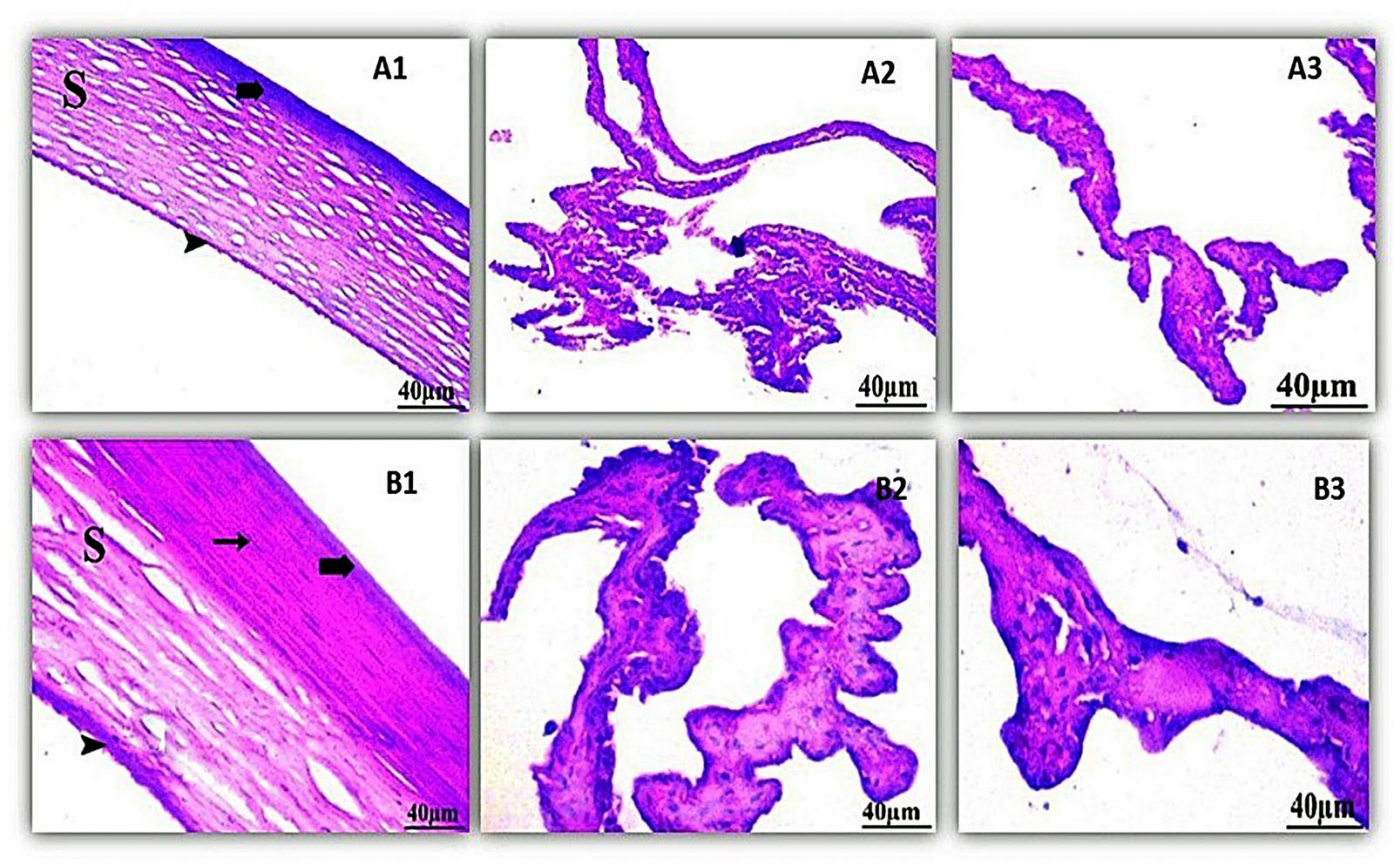
Histopathology microscopy of the cornea (1), ciliary body (2), iris (3) after treatment with the optimized formula of 21 days. Notes: (A) Control; (B) treated, (bold arrow) Showing outer epithelium, (arrowhead) inner endothelium, (S) intermediate stroma, and (arrow) keratocyte.

Histological examination, shown in Figure 8 of the control cornea, illustrated the outer epithelium, inner endothelium, and intermediate stroma, the three layers that made up the cornea. The stroma, which made up most of the cornea, was made up of collagenous fibers in continuous lamellae. Keratocytes were flattened cells found in between the stromal lamellae. The cornea's inner endothelium was composed of a single layer of flattened cells connected by a thick Descemet's membrane. Iris and the ciliary body had no symptoms of cellular invasion. The retinal pigment epithelium, the photoreceptor layer (rod and cone cells), the outer and inner nuclear layers, and the outer and inner plexiform layers were all visible in the control retina. The ganglion cell layer was made up of variously sized rounded cells.

On the other hand, the histological examination of the treated cornea had a slight effect on the eye cornea. Whereas the optimized formula had no effect on most layers of the retina where the outer and inner segments of photoreceptors were intact, the plexiform layers’ outer and inner layers were intact, without spaces between their fibers. The ciliary body showed no indications of lymphatic cellular aggression and iris of the eye were not affected by the formula. Thus, optimized LORA-NLCs Ocugel can be used safely for topical application to the eye.

#### Pharmacokinetics study in aqueous humor

As shown in Table 6, the parameters of pharmacokinetics were determined after an individual installation. (GP1) was the first group treated with LORA-NLCs Ocugel, proved the subsequent results: 1.1 ± 0.09 hr for the half-life (t_1/2_), 16.48 ± 0.31 mg/hr/L for the area under the curve (AUC_0–24_), and 1 ± 0.52 hr for (T_max_) and the corresponding peak concentration (C_max_) was 15.5 ± 0.23 mg/L.

**Table 6.** Aqueous humor pharmacokinetic parameters following ocular administration of LORA formulations.

Parameters	GP1 LORA-NLCs ocugel	GP2 pure LORA dispersion loaded in HPMC-K100 gel
t_1/2_ (h)	1.1 ± 0.09	1 ± 0.11
T_max_ (h)	1 ± 0.52	1 ± 0.21
C_max_ (mg/L)	15.5 ± 0.23	7.5 ± 0.16
AUC_0–24_ (mg/h/L)	16.48 ± 0.31	10.96 ± 0.25

While the results obtained from the second group (GP2) treated with pure LORA dispersion loaded in HPMC-K100 gel, revealed the half-life (t_1/2)_ was 1 ± 0.11 hr and the area under the curve (AUC_0–24_) was 10.96 ± 0.25 mg/hr/L, with a (T_max_) of 1 ± 0.21 hr and corresponding peak concentration (C_max_) was 7.5 ± 0.16 mg/hr/L.

Meanwhile, both (t_1/2_) and (T_max_) of (GP1) showed a non-significance difference (*P* > 0.05) compared with (t_1/2_) and (T_max_) of (GP2), on the other hand, the C_max_ was higher in the (GP1) than (GP2) with significance difference (*P* < .0001), in addition to the (AUC_0–24_), significantly increased in the (GP1) compared with (GP2) (*P* < .0001). This result might be due to the fabrication of LORA as NLCs utilizing Labrasol which is considered a nonionic water-dispersible surfactant with high HLB for lipid-based formulations that increase the bioavailability of poorly water-soluble APIs (Bertoni et al., 2020).

These findings were consistent with the higher trans-corneal penetrability of LORA-NLCs Ocugel which indicated prolonged drug retention capacity on the corneal surface compared with LORA dispersion. Additionally, these outcomes agreed with various studies, that displayed the impact of the drug encapsulation in nanoparticles on the pharmacokinetics of the drug in aqueous humor, where (Ban et al., 2017) found that lipid nanoparticle-loaded dexamethasone showed enhanced drug penetration through the corneal tissue thus, increase the ocular bioavailability compared with an aqueous solution of dexamethasone

## Conclusion

The present work indicates optimized NLCs, utilizing Design Expert, prepared using 50% of Compritol 888® to 50% of Labrasol^®^ and Span^®^ 60 that exhibited superior solubility enhancement, trans-corneal penetrability, and bioavailability of LORA the hydrophobic drug. The significant sustained effect of LOAR-NLCs was obtained through preparing LORA-NLCs Ocugel, which prolonged the residence time and the contact with the corneal surface. Furthermore, the Draize test and histological examinations confirmed the safety of optimized LORA-NLCs Ocugel for topical application into the eye. It can be concluded that LORA-loaded NLCs and their gel form represent a promising formulation for sustained release for the treatment of conjunctivitis symptoms, such as eye redness, irritation, and eye soreness thus can be safely used by COVID-19 patients and enhance the patient’s compliance.

## Supplementary Material

Supplemental Material
