# Supplementary material for: Fabrication of nanostructured lipid carriers ocugel for enhancing Loratadine used in treatment of COVID-19 related symptoms: statistical optimization, in-vitro, ex-vivo, and in-vivo studies evaluation
Source: Drug Deliv. 2022 Sep 5;29(1):2868–82. doi: 10.1080/10717544.2022.2115164 (PMC9448409; doi:10.1080/10717544.2022.2115164)
Supplement: Supplemental Material [file IDRD_A_2115164_SM5532.docx]

**Raw data for this table 4 (all statistical analysis were carried out using GraphPad Instat 3 appling student t test)**

|  | fresh | | | | |
| --- | --- | --- | --- | --- | --- |
|  | **value 1** | **value 2** | **value 3** | **Average** | **SD** |
| EE | **96.26** | **96.07** | **95.01** | **95.78** | **0.673572565** |
| PS | **155.5** | **156.52** | **156.31** | **156.11** | **0.53860932** |
| ZP | **39.463** | **40.477** | **40.36** | **40.1** | **0.554751296** |
| Q6h | **100.88** | **98.73** | **99.45** | **99.68667** | **1.094364351** |

| 4° C | | | | | T-Test |
| --- | --- | --- | --- | --- | --- |
| value 1 | **value 2** | **value 3** | **Average** | **SD** |  |
| 89.94 | **92.3** | **91** | **91.08** | **1.182032** | **sig P= 0.0039** |
| 166.37 | **167.63** | **164** | **166** | **1.843068** | **sig P= 0.0009** |
| 37.66 | **37.49** | **36.51** | **37.22** | **0.620725** | **sig P= 0.004** |
| 96.99 | **98.47** | **99.11** | **98.19** | **1.087382** | **not sig P= 0.1682** |

| 25° C | | | | | T-Test |
| --- | --- | --- | --- | --- | --- |
| value 1 | **value 2** | **value 3** | **Average** | **SD** |  |
| 88.4 | **91.35** | **92.38** | **90.71** | **2.065744** | **sig p=0.0156** |
| 163.82 | **161.27** | **163.4** | **162.83** | **1.367223** | **sig P= 0.0014** |
| 32.02 | **31.84** | **34.75** | **32.87** | **1.630613** | **sig P= 0.0019** |
| 96.22 | **98.47** | **101.68** | **98.79** | **2.74403** | **not sig P= 0.6269** |
